# Sleep Duration, Insomnia Symptoms, and Emotion Regulation among Black Women

**DOI:** 10.4172/2167-0277.1000122

**Published:** 2013-06-17

**Authors:** Christie Racine, Kaushal Kalra, Mirnova Ceide, Natasha J. Williams, Ferdinand Zizi, Mauro V Mendlowicz, Girardin Jean-Louis

**Affiliations:** 1 Center for Healthful Behavior Change, Department of Population Health, NYU Langone Medical Centre, NY, USA; 2 Department of Family Practice, SUNY Downstate Medical Centre, NY, USA; 3 Albert Einstein College of Medicine of Yeshiva University, Department of Psychiatry and Behavioural Sciences, NY, USA; 4 Department of Psychiatry and Mental Health, Universidade Federal Fluminense, NY, USA; 5 Institute of Psychiatry, Universidade Federal do Rio de Janeiro, NY, USA

**Keywords:** Sleep duration, Emotion regulation, Anxiety, Women, Race/ethnicity

## Abstract

**Introduction:**

This study explored the associations between sleep duration and emotion regulation among urban black women (mean age=59 ± 7 yrs).

**Method:**

Eligible women (n=523) provided sociodemographic data during face-to-face interviews. We used the Comprehensive Assessment and Referral Examination Physical to measure health status; women also estimated their habitual sleep duration. We utilized a modified version of Weinberger’s conceptual model of repression, the Index of Self-Regulation (ISE) to measure emotion regulation. ISE scores were derived by amalgamating the defensive subscale from the Social Desirability Scale and the anxiety subscale from the State-Trait Anxiety Inventory.

**Results:**

The median habitual sleep duration was 7 hours; 20% of the women were short sleepers (<6 hours) and 6% were long sleepers (>8 hours). Short sleepers, rather than long sleepers, had a greater likelihood of reporting insomnia symptoms than those sleeping 6–8 hours [63.4% vs. 28.1%; Χ^2^ = 41.87, p<0.001]. In the first logistic regression model, the odds of being a short sleeper for low regulators were 3 times greater than for high regulators [OR = 3.22 95% CI: 2.05–5.06; p<0.0001]. In multivariate-adjusted analysis, OR was reduced to 2.06, but remained significant. In the second logistic model, the likelihood of being a long sleeper among low regulators were 37% greater than for high regulators, but results were not significant [OR=1.37, 95% CI: 0.62–3.01; NS].

**Discussion:**

Short and long sleep duration are associated with reduced ability for emotion regulation. Women sleeping 6–8 hrs might be more adept at regulating emotions in their daily lives. Insomnia symptoms might mediate associations between emotion regulations and sleep durations.

## Introduction

Several published reports have suggested that black race/ethnicity is independently associated with extreme sleep durations [1–3]. Short sleep, defined as sleeping less than 6 hours, which is decried as a public health hazard, is more prevalent among blacks [3,4]. Long sleep, sleeping greater than 8 hours, which is often associated with psychological illnesses, is likewise more prevalent among blacks [3–5]. While epidemiologic findings favour worse physical and psychological health outcomes individuals sleeping abnormally less or more than the population mode (7 hours), [5–9] little has been done to examine what specific illnesses might be associated with short or long sleep among blacks, an understudied population regarding adverse effects of inadequate sleep durations.

There is ample literature evidencing associations between extreme sleep durations and medical co-morbidities. Epidemiologic and clinical data has indicated that short or long sleepers are at high risk for obesity,[10] diabetes, [10,11] hypertension, [10] and cardiovascular disease [10,11]. There is a dearth of published studies addressing relationships of short/long sleep durations to psychological health. This is important since psychological illnesses negatively affect the sleep process [12–15]. Generally, depression, loneliness, and stress are related to sleep [16–18] Among women, in particular, increased depression and anxiety are associated with increased sleep complaints [19] To our knowledge, there are no published studies that have specifically addressed relationships of short/long sleep durations to psychological health among blacks. In a previous study, we noted that black women exhibiting a low level of emotion regulation [20,21] were more likely to report symptoms of insomnia [22]. Unfortunately, data on sleep duration were not available permitting the determination whether blacks experiencing short or long sleep might have reduced ability to regulate their emotion.

The present study tested the hypothesis that emotion regulation would predict inadequate sleep (sleeping less than 6 hours or longer than 8 hours) among black women. Emotion regulation refers to individuals’ ability to distance themselves psychologically from events discretely appraised as negative or situations that threaten their self-concept. The present study utilized a modified version of Weinberger’s conceptual model of repression to capture individuals’ level of emotion regulation [21]. We also explored whether the presence of insomnia symptoms would mediate associations between emotion regulations and sleep durations.

## Methods

Data for the present analyses came from a community-based study assessing physical and psychological health among older adults in Brooklyn, NY. The study sample comprises 523 black women (mean age=59 ± 7 yrs). The present analyses focused on associations of short sleep (<6 hrs) and long sleep (>8 hrs) with emotion regulation.

### Procedures

Trained, quality-controlled interviewers administered several scales/questionnaires during face-to-face interviews conducted in the volunteer’s home or a location of their choosing (usually a church or a senior center). Interviews lasted approximately 1.5 hours, and women completing the study received $25 for participating. The Internal Review Board at Long Island University approved the study.

Measures for the present analysis included sociodemographic and medical factors: age, education, income, and BMI. Physical health was measured with the Comprehensive Assessment and Referral Evaluation (CARE). Repressive coping was assessed with the Index of Self-Regulation of Emotion (ISE). According to Hoyle and Bradfield, self regulation, the means by which people manage their attention, thoughts, motivation, feelings, and behavior is a unique and important predictor of consequential personal, social, and economic outcomes [23].

The CARE has been widely used to assess physical health of older individuals in minority communities. It has shown good construct validity [24] as well as concurrent and predictive validity; [25] subscales included in our analyses were: somatic complaint, sleep disorder, leg problems, heart disease, respiratory disease, arthritis, vision problems, and hypertension (Cronbach α=0.86; 0.85; 0.86; 0.83; 0.72; 0.91; 0.85; and 0.92, respectively). Additionally, women were asked to estimate habitual sleep duration (using full hour units i.e., 5 hours, 6 hours, 7 hours, etc.); no information on specific sleep disorders was elicited during the interview.

The Index of Self-Regulation constitutes a modified version of Weinberger’s conceptual model of repression [21]. According to Mendolia’s research, the model stipulates that the interaction of individual differences in emotional responsiveness and situational threats to self-concept contributes to one’s tendency to regulate emotional responsiveness. This model of repressive behavior posits that repressive copers are hypersensitive to both negative and positive emotional events, but they distance themselves from these events only when the situation threatens their self-concept [26–28]. Repressive coping refers to a person’s belief that he or she is capable of conforming to rigid standards of self-control [20].

Mendolia’s model represents an extension of conventional categorical measures of repression, which might not yield an accurate representation of observed variations in repressive coping [26]. This revised model accounts for the motivation and conditions in which repressors use a perceptual defense in response to negative and positive emotional events. In our analysis, ISE scores were derived following Mendolia’s conceptualization, which amalgamates the defensive scale of the Social Desirability Scale (α=0.73) [20] and the anxiety subscale of the State-Trait Anxiety Inventory (α=0.75). [29] Scores ranged from 0 to 52, with higher scores representing greater defensiveness/repressive coping. According to this classification scheme, individuals have a repressive coping style when they are highly defensive (e.g., high score on the social desirability) scale, but also low in trait anxiety (e.g., low score on the manifest anxiety scale). Details on the derivation of ISE scores as used in our analysis have been reported elsewhere.[30

The stress index scale used initially by the National Survey of Black Americans was administered to our participants [31]. Respondents rated on a 4-point scale the degree to which a set of items provoked stress in the past month or two. These stress-induced life events were health, money, job, and problems with family or marriage, problems with people outside the family, children, crime, police, love life, and racial conflict. Scores ranged from 0 to 29, and higher scores denoted greater stress levels (α=0.81).

### Statistical Analysis

To describe the sample, we used frequency and measures of central tendency. In preliminary analyses, Pearson and Spearman correlations were used to explore relationships between variables of interest. Chi’s square test was employed to assess differences in categorical variables. For descriptive purposes, the sleep measure classified participants into three groups: those reporting short sleep (<6 hrs) or long sleep (>8 hrs) referenced to those reporting sleeping 6 to 8 hours. These cut-off points were chosen based on previous research showing health risks associated with short and long sleep durations [32–35]. The Index of Self-Regulation of emotion was dummy-coded as high regulator vs. low regulator using a median split.

To test the hypothesis that emotion regulation was associated with short sleep [<6 hrs] and long sleep [>8 hrs], we utilized logistic regression modeling. For both models: A) short sleep and B) long sleep, first we performed univariate logistic regression analyses to determine associations of emotion regulation with short or long sleep. Next, we performed multivariate logistic analyses to ascertain likely effects of several covariates including age, education, income, BMI, and a history of hypertension, respiratory disease, arthritis, heart disease, and vision problems. We also assessed the contribution of insomnia symptoms (i.e., difficulty initiating sleep, difficulty maintaining sleep and/or early morning awakening) in the logistic models. Before constructing the models, we assessed bivariate associations between hypothesized predictors and the dependent variable; only factors showing a p value <0.05 were entered in the final model [36]. All analyses were performed using SPSS 18.0.

## Results

Of the sample, 80% had at least a high school diploma. The median income of those women was $10,000, and the median BMI was 29 kg/m^2^. Thirty-eight percent of the women reported heart disease; 66%, arthritis; 23%, respiratory disease; 60% hypertension; and 50%, vision problems. Thirty-six percent reported insomnia symptoms: difficulty initiating sleep, difficulty maintaining sleep, or early morning awakening. The median habitual sleep time was 7 hours; 20% of the women were short sleepers (<6 hrs) and 6% were long sleepers (>8 hrs). In table 1, we compare the total number of women reporting differing sleep durations based on their classification as a low or high regulator.

Using Chi square tests, we found that women sleeping less than 6 hours habitually had a greater likelihood of reporting insomnia symptoms than those sleeping 6–8 hours [63.4% vs. 28.1%; Χ^2^ = 41.87, p<0.001]. We observed no significant difference in the likelihood of experiencing insomnia symptoms between women sleeping longer than 8 hours and those sleeping 6–8 hours [32.1% vs. 32.0%; Χ^2^ = 2.87, NS].

We constructed two logistic regression models. Results of the first logistic regression model showed that the odds of being a short sleeper for low regulators were 3 times greater than for high regulators [OR=3.22 95% CI: 2.05–5.06; p<0.0001]. As shown in table 2, in multivariate-adjusted analysis OR was reduced to 2.06, but remained significant; differences in age, education, income, BMI, stress level, and a history of hypertension, heart disease, respiratory disease, arthritis, and vision problems were adjusted. Results of the second logistic model showed that the likelihood of being a long sleeper among low regulators was 37% greater than for high regulators; however, results were not significant [OR=1.37, 95% CI: 0.62–3.01; NS]. Results for each factor entered in the multivariate logistic models are shown in table 2.

As expected, when the insomnia factor was added to the short sleep model, the odd associated with being a short sleeper for low regulators was significantly reduced [OR=1.44, 95% CI: 0.82–2.52, NS]. We did not explore the addition of the insomnia factor to the long sleep model, as results of the initial model were not significant.

## Discussion

Linking sleep with emotion regulation is not a novel concept. Many studies have shown that those two factors are intimately related, likely in a bidirectional fashion [37,38]. Although emotion regulations may vary across the lifespan, [39,40] relationships with sleep persist [37]. In the biological sciences, recent data on the interaction between sleep and emotion regulation has provided evidence supporting the view of a sleep-dependent emotional brain processing [41,42]. With emerging interest in the associations of emotions with sleep, a question has arisen as to the possible effects of reduced sleep time on the ability to regulation emotions, since sleep duration has declined over the years [5–9]. The present study demonstrates that women sleeping less or more than the population average sleep time (6–8 hours) might experience difficulty regulating their emotions.

The main finding of our analysis is that women classified as low regulators were at increased odds of being short or long sleepers, relative to women exhibiting a high level of regulating their emotions. Specifically, our first regression model showed that the odds of being a short sleeper for low regulators were 3 times greater than for high regulators. Likewise, our second logistic model showed that there was a 37% greater likelihood for low regulators to report sleeping longer than the average sleep time. Of note, when we adjusted for differences in sociodemographic and medical factors, the model explaining associations of emotion regulation with short sleep remained significant, although the adjusted odds ratio was reduced (OR=2.06). That the model for long sleep was not significant, even before the inclusion of confounders, may be attributed to lack of statistical power to detect significance associations. Only 6% of the women reported long sleep durations, whereas 20% indicated short sleep durations.

As reduced sleep time is often associated with the presence of insomnia, we performed a separate analysis adjusting for potential effects of insomnia symptoms on relationships between emotion regulation and short sleep duration. In the present study, 36% of the women reported insomnia symptoms (i.e., difficulty initiating sleep, difficulty maintaining sleep, or early morning awakening). As expected, when the insomnia factor was added to the short sleep model, the likelihood of being a short sleeper for low regulators was 44% greater than for high regulators. These findings suggest that much of the effect of emotion regulation on short sleep seems to be mediated by the presence of insomnia symptoms. This is consistent with our previous analysis of community-based physical and psychological health data obtained from 1,274 women [22]. That analysis showed significant associations between emotion regulation and insomnia symptoms for both black [r_s_=−0.43, p<0.0001] and white women [r_s_=−0.18, p<0.0001]. Hence, although short sleep is independently associated with emotion regulation, one cannot discount the presence of insomnia symptoms in analysis examining effects of emotion regulation on short sleep durations.

Interestingly, we observed no significant difference in the likelihood of experiencing insomnia symptoms comparing women sleeping longer than 8 hours and those sleeping 6–8 hours. There is evidence suggesting that some individuals classified as long sleepers may experience insomnia symptoms, resulting from various medical [43,44] and psychological conditions [45,46]. It is of interest to examine effects of emotion regulation on long sleep duration in larger samples, providing data allowing adequate statistical control.

Our focus on individuals of black race/ethnicity was guided by data evidencing that short and long sleep durations are more prevalent among blacks, [3] and that blacks reporting sleep problems have significantly poorer physical health than other ethnic groups [47]. Future studies should investigate whether associations between emotion regulation and sleep durations would be similar for women from other racial/ethnic groups. In our previous study, we found that women of black race/ethnicity exhibited greater ability to regulation their emotions, relative to white women [22]. This differential racial/ ethnic effect, in part, explained why black women reported fewer insomnia complaints than their white counterparts.

Our study has notable limitations. First, since we used an observational design, we could not establish the mechanism by which emotion regulation is associated with short or long sleep duration. Second, we did not use a population-based representative sample of US adults. Thus, our results are not generalizable to other racial/ethnic groups. Notwithstanding these limitations, our study has several strengths. Many studies in the past have not specifically examined associations between emotion regulation and sleep durations, particularly among black women, an understudied population. Strength of our study is that it examines association of emotion regulation with both short sleep and long sleep; previous studies have not examined long sleep as a correlate of emotion dysregulation. Future studies should explore those associations among Hispanic and Asian populations as well.

## Figures and Tables

**Table 1:** Comparison of low regulators and high regulators based on reported sleep duration; values represent number and percent of cases for each sleep category.

Self-Reported Sleep Duration Based on Level of Emotion Regulation (n = 523)		
Sleep	Emotion Regulation	
	Low Regulator (38%)	Hi Regulator (62%)
≤ 5 hours	60 (59.4)	41 (40.6)
6 hours	32 (38.6)	62 (61.4)
7 hours	42 (30.2)	97 (69.8)
8 hours	46 (29.9)	108 (70.1)
≥ 9 hours	11 (39.3)	17 (60.7)

**Table 2:** Regression coefficients of the sleep duration measure on emotion regulation (High Regulator vs. Low Regulator), sociodemographic factors, and medical factors. The sleep measure included individuals who reported short sleep durations (<6 hrs) or long sleep (>8 hrs) vs. those sleeping 6–8 hours habitually;

Associations of Emotion Regulation, Sociodemographic, Medical Factors with Sleep Duration				
	Model A: Short Sleep		Model B: Long Sleep	
Variables	OR	95% C.I.	OR	95% C.I.
**Low Emotion Regulation**	2.06**	1.22–3.48	1.87	.74–4.75
**Age**	1.04	.99–1.08	.92	.86–.99
**Education**	1.11	.82–1.49	1.38	.76–2.52
**Income**	1.00	.99–1.02	1.00	1.00–1.02
**Stress**	1.01	.97–1.05	1.10*	1.01–1.19
**BMI**	1.00	.96–1.04	.96	.89–1.04
**Heart Disease**	.57	.32–1.02	.67	.25–1.96
**Hypertension**	1.41	.82–2.43	.83	.32–2.17
**Respiratory Disorders**	.61	.35–1.06	1.11	.35–3.58
**Arthritis**	.88	.49–1.58	1.37	.49–3.77
**Vision Problems**	.53	.29–.99	2.63	.92–7.48

** p<0.001.

* p<0.05.
